# Superlatives, clickbaits, appeals to authority, poor grammar, or boldface: Is editorial style related to the credibility of online health messages?

**DOI:** 10.3389/fpsyg.2022.940903

**Published:** 2022-08-29

**Authors:** Katarína Greškovičová, Radomír Masaryk, Nikola Synak, Vladimíra Čavojová

**Affiliations:** ^1^Faculty of Social and Economic Sciences, Institute of Applied Psychology, Comenius University in Bratislava, Bratislava, Slovakia; ^2^Centre of Social and Psychological Sciences, Institute of Experimental Psychology, Slovak Academy of Sciences, Bratislava, Slovakia

**Keywords:** message credibility, adolescents, scientific reasoning, analytical thinking, media literacy

## Abstract

Adolescents, as active online searchers, have easy access to health information. Much health information they encounter online is of poor quality and even contains potentially harmful health information. The ability to identify the quality of health messages disseminated *via* online technologies is needed in terms of health attitudes and behaviors. This study aims to understand how different ways of editing health-related messages affect their credibility among adolescents and what impact this may have on the content or format of health information. The sample consisted of 300 secondary school students (*M*_*age*_ = 17.26; SD_*age*_ = 1.04; 66.3% female). To examine the effects of manipulating editorial elements, we used seven short messages about the health-promoting effects of different fruits and vegetables. Participants were then asked to rate the message’s trustworthiness with a single question. We calculated second-order variable sensitivity as the derivative of the trustworthiness of a fake message from the trustworthiness of a true neutral message. We also controlled for participants’ scientific reasoning, cognitive reflection, and media literacy. Adolescents were able to distinguish overtly fake health messages from true health messages. True messages with and without editorial elements were perceived as equally trustworthy, except for news with clickbait headlines, which were less trustworthy than other true messages. The results were also the same when scientific reasoning, analytical reasoning, and media literacy were considered. Adolescents should be well trained to recognize online health messages with editorial elements characteristic of low-quality content. They should also be trained on how to evaluate these messages.

## Introduction

In today’s digital world we are faced with vast amounts of information generated by social networks and the internet at large, in addition to traditional outlets. “The era of fake news” (Albright, 2017: 87) and the “information pandemic” challenge us in several ways and it has become quite hard to navigate in the realm of news and information.

Extremely easy access to health advice (such as social media for information on nutrition, Poínhos et al., 2017) is not a guarantee that the information encountered is valid and helpful. Indeed, recent research has confirmed that online health messages are mostly unsatisfactory, incomplete, and inaccurate, and/or have misleading content or even potentially harmful health information (e.g., Dutta et al., 2020, on COVID-19 and severe acute respiratory syndrome coronavirus 2; Goobie et al., 2019, on idiopathic pulmonary fibrosis; Loeb et al., 2020, on urological health issues; Mueller et al., 2019, on psoriasis; Mueller et al., 2020, on atopic eczema). Consequently, trusting health messages of an unsatisfactory quality can result in poor health choices that are ineffective at best and lethal at worst. For instance, conspiracy beliefs can have a real effect on general health. Recently published research showed that belief in conspiracy relates to negative attitudes toward vaccination (Jolley and Douglas, 2017: 459; Hornsey et al., 2018: 310–311). Thus, trustworthiness of online messages has become an issue of utmost importance (Flanagin and Metzger, 2008).

Trusting in a message or message credibility could be defined as “an individual’s judgment of the veracity of the content of communication” (Appelman and Sundar, 2016: 63). Hence it refers to the perceived credibility and trustworthiness of the information, not the measure of the actual quality of the information (Freeman and Spyridakis, 2004: 240). Message credibility is also distinct from media or source credibility (e.g., Flanagin and Metzger, 2007; Appelman and Sundar, 2016; Sungur et al., 2016), however, it could be complicated to separate one from the other when evaluating message (Brante and Strømsø, 2018: 794). Often, judging source credibility is a pre-step in further assessing message credibility (Flanagin and Metzger, 2008).

Message credibility can be evaluated by describing its accuracy, authenticity, and believability (Appelman and Sundar, 2016: 73). The last one is considered to be the overarching characteristic of message credibility (Flanagin and Metzger, 2008) and, therefore, we wanted to examine this characteristic of health messages and decided to focus on the perceived persuasive intent of messages, which is one of the five heuristics people use when assessing online messages (Metzger et al., 2010: 434–435). The message itself can possess some features that contribute to higher perceived credibility, such as statistics and references (Hong, 2006: 551).

Prior work on message credibility has been interdisciplinary, such as information research or social psychology (Brante and Strømsø, 2018: 774–778). Typical research focuses on distinguishing fake and true information and on the different cognitive processes involved in the evaluation (e.g., Pennycook et al., 2020). Other branches of research deal with factors influencing message credibility, such as content or format features. For example, research shows that the strength of arguments is the main factor in message assessment (Li and Suh, 2015: 323). But this path of research is usually overlapping with source credibility research.

Since most of the research on credibility messages involves adult samples (e.g., Newman and Fletcher, 2017; Sterrett et al., 2018), this research focuses on adolescents as being “digital natives” (e.g., Bennett and Maton, 2010). However, a recent systematic analysis expresses concern about little research in this area and its possible impact on adolescents’ health and life (Freeman et al., 2020: 215). Youth is an especially sensitive period for the development of good health practices, and it seems particularly salient to support healthy lifestyle preferences in this period (Kelly et al., 2011: 220–221). For instance, recent research has shown that risky behavior in youth is promoted by positive portrayals in the media, such as exposure to pro-smoking portrayals in movies (Sargent et al., 2005: 1185–1186) or to pro-alcohol portrayals (Hanewinkel et al., 2012: 712–714). Information obtained from interpersonal, online, or media sources changes how one approaches health and illness (Bell, 2014: 514). Online health information supports information provided by practitioners and participants report positive effect on health-related decisions, such as lifestyle changes, self-care, treatment compliance (Thapa et al., 2021: 780–781).

We cannot expect the media to provide healthier content or to drop unhealthier messages, it is up to the readers how they approach the messages (Brown, 2006: 459). They usually evaluate online information based on website appearance, website domain, and website language (Freeman et al., 2020: 219–220), meaning that they look at their structural features (Rains and Karmikel, 2009: 549–551). Website messages that use business-like language, authoritative organizations, or trusted brands are more trusted (Freeman et al., 2020: 219–220). Based on adolescents’ reports in focus groups, they are also sensitive to editing elements (superlatives, clickbait, grammar mistakes, authority appeal, and bold typeface). Erroneous or insufficient editing influenced the distinction between the credibility and the untrustworthiness of messages (Vorelová and Masaryk, 2019).

Even though adolescents use online health information immensely, adolescents’ strategies to appraise the information are not sophisticated (Freeman et al., 2020: 219). Adolescents search less systematically (Bilal and Kirby, 2002: 661–663; Hansen et al., 2003: 7), their search is superficial, and they seldom consider the source of the information (Hansen et al., 2003: 7; Wallace et al., 2000: 93–94). It seems that adolescents’ skills are rather inadequate and insufficient (Marttunen et al., 2021: 300–301). When evaluating sources, their assessments are focused more on relevance or accessibility (non-epistemic justification) than reliability or credibility (epistemic justification) (Coiro et al., 2015: 294; List et al., 2016: 47). And even if they find the proper information, secondary school students cannot reliably distinguish sponsored content in the text from the original editorial content (Wineburg et al., 2016). They usually believe what they encounter online, regardless of the marketing practices (Kim et al., 2011: 195). Therefore, it is very important to understand which health messages encountered online are trustworthy, and relevant, without commercial backgrounds such as advertising, promotion, or sponsorship (Kim et al., 2011: 188).

Most of the research on credibility messages involves adult samples (e.g., Newman and Fletcher, 2017; Sterrett et al., 2018). However, adolescents are active users and searchers for online health information. The relevance and quality of this information are questionable, we need to examine which information they perceive as credible. Although previous research has explored the strategies for judging online information, most of the research does not separate message credibility from media credibility, and little is known about message credibility and its prominent part- believability (Appelman and Sundar, 2016: 59–60). Since most of the studies concerning media trust are cross-sectional (Strömbäck et al., 2020: 146–147), we wanted to understand how manipulations with health messages affect their trustworthiness. Previous research has also suggested that both format and content of online messages might be important in perceiving messages as trustworthy (Metzger et al., 2003: 302–304). This study aims to understand how manipulations with short health messages affect their trustworthiness in adolescents and what implications this may have on content or format of health information. Moreover, this study builds on the previous study with high school students (Vorelová and Masaryk, 2019), in which the authors qualitatively explored message trustworthiness and identified five editing elements that deduced perceived message credibility. Accordingly, we wanted to experimentally verify the effect of content and format manipulations of short health messages (fake health messages, true health neutral messages, and true health messages with editing elements) on trust in messages. We hypothesized that adolescents perceive true messages as more trustworthy than fake messages (RH1). We also wanted to know how well the participants discern between true neutral messages and fake messages. Therefore, we explored their sensitivity to these messages, inspired by bullshit sensitivity (Pennycook et al., 2015). Sensitivity was calculated as deduction of the fake message score from the true neutral message score. Our second hypothesis thus stated that our participants are more sensitive to true neutral messages than to fake messages (RH2). We also hypothesized that true neutral messages will be more trusted than messages with editing elements (RH3) Lastly, we hypothesized that messages with editing elements are more trusted than fake messages (RH4).

However, the perception of message credibility can depend on previous knowledge and skills of participants. For example, previous research has shown that analytical thinking, scientific reasoning, and media literacy might help high school students to discern between trustworthy and untrustworthy messages. Analytical thinking is considered to be a broader cognitive ability, while scientific reasoning is a narrower ability. Analytical thinking in the dual-process theory (Kahneman, 2019) or Type 2 processing in the tripartite model of the mind (Stanovich et al., 2011: 374) are defined as conscious, effortful, slow, logical, and systematic. It is usually compared to intuitive thinking or System 1, which is described as fast, effortless, uncontrolled, and emotional. When a problem is simple, intuitive thinking helps us to come to quick decisions, gives us important cognitive shortcuts, and thus reduces endeavor and time. Analytical thinking is best suited for complex and complicated problems. Several recent studies (e.g., Pennycook and Rand, 2019; Pennycook et al., 2020) showed that people with better analytical thinking are usually better at discerning fake news from the real news, regardless of their political orientation. These studies examined mainly political fake news and used adult samples; therefore, we aim to examine the protective role of analytical thinking against manipulation of the messages in high school students (RQ1).

However, recent studies showed that besides analytical thinking, scientific reasoning is important in credibility issues as well (e.g., Čavojová and Ersoy, 2020). Scientific reasoning is defined as an ability to understand and apply principles of sciences, such as understanding hypotheses and theories, gathering data, and examining the evidence (Dunbar and Klahr, 2012). Research shows that scientific reasoning is related to distrust of alternative treatments and pseudoscientific health practices (Čavojová and Ersoy, 2020: e244). Scientific reasoning also predicted endorsement of general as well as health specific (related to COVID-19) unwarranted beliefs over and above analytical thinking (Čavojová et al., 2019: 5–7; Čavojová et al., 2020: 543–544). Therefore, to examine its effect on the manipulation of the messages we included scientific reasoning in our study (RQ2). Since domain-specific knowledge is bound to concrete problems that can be easier handled for adolescents (Bašnáková and Čavojová, 2019), we applied concrete scientific reasoning in the study.

Lastly, not everybody is motivated or able to reason analytically and scientifically, thus the focus is shifted more and more toward second-order scientific reasoning, such as media literacy. For instance, it helps in reducing current smoking and susceptibility to future smoking (Primack et al., 2006: 469) as well as to reduce the consumption of sugar-sweetened-beverages (Chen et al., 2017: 7). Furthermore, media literacy interventions decrease deviant behavior such as alcohol intake, smoking, body dissatisfaction, and eating disorders (Xie et al., 2019:153). The current study, therefore, explored whether media-savvy adolescents would be affected by manipulation of the messages in information trust (RQ3).

## Materials and methods

### Research design

We used the experiment with an incomplete within-subject repeated design with 1 factor (message) in 7 levels (fake message, true neutral message, true message with editing elements- superlatives, clickbait, grammar mistakes, authority appeal, bold typeface). Each participant randomly received every level of the independent variable (fake message, true neutral message, true messages with editing elements- superlatives, clickbait, grammar mistakes, authority appeal, bold typeface). We also ensured that each level of the independent variable (except fake message) was displayed with different content (different fruit or vegetable). The randomization of the independent variable was ensured by the Qualtrics program. There were three covariates to control the effect of the manipulation on the trustworthiness. No blinding was involved in this study.

### Participants

The G*power 3.1 (RRID:SCR_013726) with the defined parameters calculated the required sample size to be 282. In the experiment, the target sample size was 300 including participants who might fail to pass the attention check question. The data collection agency stopped when *N* = 300 was reached.

The online research was opened by 721 participants, out of which 279 withdrew at some point, 98 finished the experiment before getting to the attention check question, 32 were excluded based on the results of the control question, and 12 did not finish the experiment after the control question. In total, 300 participants successfully finished the experiment. Participants that responded incorrectly to attention check question were excluded. There were no incomplete or missing data. We set up items in the program as obligatory. Only full answers were analyzed.

The sample consisted of respondents who attend secondary school in Slovakia aged 16–19 years. Respondents were informed of the intention and rules of the research through informed consent. Respondents were recruited *via* a research agency. The agency addressed respondents who met the following conditions: high school students aged 16–19, even distribution of men/women, and coverage from all regions of Slovakia. All respondents who required it were recruited by the agency through their legal representatives, who gave their consent for them to participate in the research. The research agency used an online panel to address respondents or their legal representatives.

The sample consisted of 66.3% of women, *M*_*age*_ = 17.26 (SD = 1.04). In total, 36.7% of students attended secondary grammar school, and 63.3% of students attended various specialized types of high school.

### Materials

The research battery consisted of the presentation of short health messages, and the evaluation of their trustworthiness, followed by a single attention check question (out of 4 possibilities participants were asked to tick one). This was followed by three instruments measuring scientific reasoning, analytical thinking, and media literacy, respectively.

#### Experimental manipulation of the editing elements in short health messages

##### Health messages

To study the effect of manipulation of editing elements, we used six short messages (up to 35 words) about the beneficial health effects of various fruits and vegetables. The messages were based on real research findings and the example of the structure was: *“Natural source of pectin (Title). Carrot has a positive effect on cholesterol. It is because carrots contain pectin, which helps with decreasing cholesterol levels. Pectin enables exclusion of toxic substances from the body.”* (The exact wordings of all used messages).^1^ The six messages were chosen based on the results of the pilot study (see text footnote 1) to reflect the same level of trustworthiness.

Five new (manipulated) versions of each message were then created: (1) including 3 superlatives in the text of the message, (2) using clickbait title (3) including 3 small grammar mistakes in the text of the message, (4) adding a fictional source of the message^2^, and (5) using 3 boldfaces to emphasize several words in the message. This created a pool of 36 messages (six messages in six versions, available at (see text footnote 1).

To examine the ability to distinguish between true and fake health messages, we also included one fake message in the same format as true neutral health messages: *“Natural source of iqmerctin (Title). Beans have a positive effect on intelligence. They contain a substance called iqmerctin, which helps to increase intelligence. Iqmerctin is one of the active ingredients supporting brain development.”* We used a made-up substance called “iqmerctin.” The word is an amalgamation of the abbreviation IQ and word ivermectin which at the time of data collection was considered controversial in relation to treating COVID-19. The antiparasitic drug was initially considered to be a COVID-19 miracle cure but later research did not support the claim (Popp et al., 2021).

#### Dependent variable

##### Trustworthiness

Participants were asked to assess the trustworthiness of the messages by answering the question “*To what extent do you believe this message*?” on a Likert scale ranging from 1 (*not at all trustworthy*) to 5 (*totally trustworthy*). A single score was used for each message (theoretical range from 1 to 5) with higher numbers indicating higher trust in the message.

As a secondary variable, we also calculated the ability to recognize true and fake messages. Second-order variable sensitivity was calculated as a deduction of trustworthiness in fake messages from the trustworthiness of true neutral messages. The theoretical range is from −5 to +5. Positive numbers indicated higher trust in true neutral messages than fake messages, negative numbers the opposite. This allowed us to see the level of trust participants placed in each message and also their sensitivity to fake messages.

#### Covariates

##### Scientific reasoning

We used the Scientific Reasoning Scale (Drummond and Fischhoff, 2017) adapted by Bašnáková et al. (2021). It contains six items (available at https://osf.io/7vjxd/); for example, the “causation vs correlation” item was about increasing the birth rate: “*A researcher wants to find out how to increase natality. He asks for statistical information and sees that there are more children born in cities that have more hospitals. This finding implies that building new hospitals will increase the birth rate of a population. Agree/Disagree*.” Each correct answer was assigned 1 point and we calculated the total score as the sum of all correct answers (theoretical range from 1 to 6), thus a higher number indicates better scientific reasoning. The mean score for the whole sample was 3.93 (SD = 1.45). Cronbach’s α was 0.44 and ω = 0.45. We calculated average correct answers as the ratio of mean score and number of items. In total, 65.5% of items were answered correctly.

##### Analytical thinking

We used four items from the cognitive reflection test – two items from the numerical version (Frederick, 2005) and two items from the verbal version (Sirota et al., 2020). For example: “*If you were running a race, and you passed the person in 2nd place, what place would you be in now?*” (correct answer: 2nd, intuitive answer: 1st). The test is presented in full at https://osf.io/7vjxd/. The Sum of total correct answers (theoretical range from 0 to 4) reflects participants’ analytical thinking with higher scores indicating better analytical thinking. The mean score for the whole sample was 1.52 (SD = 1.04), Cronbach’s α was 0.38 and ω = 0.41. We also calculated average correct answers as in the previous instrument and 38% of items were answered correctly.

##### Media literacy

We will use the Critical Thinking about Media Messages scale (Scull et al., 2010) to assess media literacy. The six items of the scale (for example “*I think about what the people who made the media message want me to believe,”* presented in full at https://osf.io/7vjxd/) are evaluated on a 6-point scale from 1 (*never*) to 6 (*always*). A total score (theoretical range from 6 to 36) was calculated as the sum of all item responses. Higher scores indicate a greater frequency of critical thinking about media messages. The mean score for the whole sample was 21.65 (SD = 6.22) and both α and ω were 0.85.

### Ethical considerations

All the study’s participants provided informed consent, and the study design was approved by the appropriate ethics review board. All procedures performed in the study followed the ethical standards of the 1964 Helsinki declaration and its later amendments and the Internal Institutional Regulation 5/2014. A local ethics committee at Comenius University, Faculty of Social and Economic Sciences, upon the Regulation 5/2014 ruled that no formal ethics approval was required in this particular case.

### Data analysis

The data were analyzed using the IBM SPSS Statistics 20 (IBM SPSS Statistics, RRID:SCR_016479) and JASP software (0.12.2, RRID:SCR_015823). Descriptive analysis, Cronbach’s alpha, McDonald’s omega, paired *t*-tests, and repeated measures ANCOVA with *post-hoc* test were used to analyze the data. A *p*-value <0.05 was used as a criterion to determine the statistical significance of all analyses conducted in this study. Effect size *Cohen’s d* was interpreted based on Cohen’s suggestion (Cohen, 1992). Raw data are stored at osf.io/7vjxd.

## Results

### Sensitivity to messages

We used frequency analysis to explore the sensitivity between true neutral and fake messages (RH1, Table 1). In total, 40.7% of participants did not differentiate between fake and true health articles. The second-largest frequency (25.3%) refers to one point difference between the trustworthiness of a true health article and a fake article, which means a true health article was assessed by participants as only one point more trustworthy than a fake health article. When we add up the frequency of negative numbers of sensitivity, we see that 11% of participants trusted more fake messages than true messages. On the other hand, 48.3% of the participants trusted more health messages than fake messages. Mean of the sensitivity is close to one (*M* = 0.73; SD = 1.35). Sensitivity did not correlate with media literacy, *r*_(292)_ = 0.05, *p* = 0.409. However, it weakly correlated with scientific reasoning *r*_(292)_ = 0.17, *p* = 0.003, and analytical thinking *r*_(292)_ = 0.12, *p* = 0.032.

**TABLE 1:** Sensitivity between true neutral and fake messages.

	−4	−3	−2	−1	0	1	2	3	4	Total
Frequency	1	2	4	26	122	76	32	24	13	300
Percent	0.3	0.7	1.3	8.7	40.7	25.3	10.7	8.0	4.3	100

### Examining the effect of manipulation

Descriptive statistics for the different versions of the messages are displayed in Table 2. Based on measures of central tendency, variability, and distribution we concluded that the variables are normally distributed. To examine the effect of our manipulations on the trustworthiness of messages (RH1, RH3, RH4) we performed a series of paired samples *t*-test (Table 2).

**TABLE 2:** Paired *t*-test comparing trustworthiness between true neutral/fake health message and health messages with editing elements.

			Comparison with the true neutral health message			Comparison with the fake health message		
	*M*	SD	*t*	*p*	*d*	*t*	*p*	*d*
True neutral health message	3.52	1.13	–	–	–	9.43	<0.001	0.77
Superlatives	3.42	1.14	1.43	0.154	0.12	−7.82	<0.001	0.64
Clickbait	3.26	1.15	3.43	0.001	0.28	−6.11	<0.001	0.50
Authority appeal	3.60	1.11	−0.98	0.327	−0.08	−9.87	<0.001	0.81
Grammar mistakes	3.45	1.08	0.95	0.346	0.08	−8.91	<0.001	0.73
Bold typeface	3.45	1.11	0.87	0.385	0.07	−8.12	<0.001	0.66
Fake health message	2.79	1.21	9.43	<0.001	0.77	–	–	–

The results showed that there was a significant difference in trustworthiness between neutral health message and fake health message, *t*_(299,1)_ = −9.428, *p* < 0.001 with Cohen’s *d* = 0.77 (medium effect size). It seems that our participants were able to successfully distinguish between the true neutral and blatantly false messages. This finding was corroborated also by the results from the comparison of the trustworthiness of the fake health message with the true messages, but with editorial elements aimed to manipulate their trustworthiness. The results showed significant differences in trustworthiness between fake health messages and all other health messages with editing elements (see Table 2) with Cohen’s *d* number at least 0.50.

On the other hand, when we examined the differences in trustworthiness between true neutral messages and messages with editing elements, we found only one significant difference between true neutral health messages and health messages with clickbait, *t*_(299,1)_ = 3.429 *p* = 0.001 with Cohen’s *d* = 0.28 (small effect size). There were no other significant differences (Table 2).

### Examining relationships with scientific reasoning, analytical thinking, and media literacy

To examine the effect of covariates (scientific reasoning, analytical thinking, and media literacy) on the trustworthiness and untrustworthiness of messages (RQ1, RQ2, RQ3) we performed a series of repeated measures ANCOVA tests.

There was not a significant effect of health messages on their trustworthiness after controlling for either scientific reasoning, *F*_(1,298)_ = 3.278, *p* = 0.053 or media literacy *F*_(1,298)_ = 0.054, *p* = 0.817; but there was a significant effect of type of health message on trustworthiness of the message after controlling for analytical thinking, *F*_(1,298)_ = 5.041, *p* = 0.017. However, Bonferroni’s multiple comparisons indicated that there was a statistically significant difference in means between fake messages and both true health messages and true health messages with editing elements when controlling for scientific reasoning (*p* < 0.001), analytical thinking (*p* < 0.001), and media literacy (*p* < 0.001). There was also a statistically significant difference in means between true neutral messages and messages with clickbait when controlling for scientific reasoning (*p* = 0.014), analytical thinking (*p* = 0.013), and media literacy (*p* = 0.014).

## Discussion

### Interpretation of the results

Although adolescents search for health information online besides using other sources (such as doctors, parents, and peers), little is known about which online information they perceive as credible. Especially nowadays, in the times of not only fake news but also news that is insufficient, questionable, and potentially hazardous to health, skills and knowledge of assessing the credibility of online health messages are essential and necessary. To avoid the drawbacks of cross-sectional research and to have more insight into credibility itself, this study experimentally examined the effects of manipulation with content and format of health online messages on their trustworthiness in an adolescent sample.

Adolescents in our sample were able to discern between fake health messages and health messages whether true or slightly changed with editing elements. However, this result regarding the ability to discern the messages that are on the opposite truth scale (fake vs. true) is more complex. We examined the sensitivity to distinguish between blatant fake health messages and true health messages. It was computed as the deduction of scores on the trustworthiness of fake health messages from true health messages. The mean was low and around zero (*M* = 0.73, SD = 1.35). From deeper frequent analysis, we found that 48% of participants trusted the true neutral health messages more than the fake ones. However, 41% of participants considered fake and true neutral messages equally trustworthy and 11% considered true neutral health messages less trustworthy than fake health messages. This result is not insignificant. Putting trust in messages requires identification of fake vs. true content. Fake content could also have the form of lies and deception, and relevant research confirms difficulties in discerning lies from true statements (e.g., Hartwig and Bond, 2014). Chances of identifying lies are close to random distribution (from 50 up to 70%), regardless of whether the setting is laboratory or real-life situations, regardless of whether participants are lay people or professional lie catchers (Aamodt and Custer, 2006: 9; Bond and DePaulo, 2006: 226; Hartwig and Bond, 2014, par: Alternative metrics). Furthermore, fake or lie identification could be medium bounded. Online communication is asynchronous, detached, non-interactive, low in richness, therefore there are less informational cues present which might impede a reader to decide on truthfulness (Carlson et al., 2004: 11–13; Burgoon et al., 2005: 2). Identification of untruth content is rather a difficult process than a simple one, and various cues (verbal linguistic and verbal content, non-verbal, contextual) or meta-cues (interactions between cues) help in true vs lie decision (Carlson et al., 2004: 7–10).

In the case of health messages that seem plausible, reasonable, and probable enough, adolescents could not tell the difference between true neutral health messages and health messages with editorial elements. The results were the same when we controlled for individual differences (scientific reasoning, analytical thinking, and media literacy). It suggests that adolescents perceive these messages as trustworthy regardless of the various content and format manipulations (superlatives, appeal to authority, boldface, grammatical errors), regardless of their reasoning skills and media literacy. The only health message with an editorial element that was significantly less trusted compared to a true health message was a clickbait headline message.

It seems that in the case of health messages that seem possibly true and believable, adolescents do not either notice or decide on their trustworthiness based on editing cues (except clickbait). These editing cues (superlatives, authority appeal, bold type, and grammar mistakes) were identified by focus groups (Vorelová and Masaryk, 2019) as being the ones that are spotted in text and decrease the credibility of the message. They are rather easily noticed cues compared to other cues that need content-demanding skills or other assessment skills that adolescents might not possess. The participants rather ticked their answers without prior deeper consideration of the content or the format cues. Only clickbait seems to be “popular” enough that adolescents were able to recognize it. Clickbait headlines seem to discourage readers and may lower message credibility (e.g., Molyneux and Coddington, 2020; Kaushal and Vemuri, 2021; Molina et al., 2021).

All four factors—source, message, media, and readers— might influence overall perceived trustworthiness (Hocevar et al., 2017). In this research, messages were stripped of source and media factors. Thus, the participants could not rely on cognitive heuristics that are frequently used to assess online messages (e.g., Metzger et al., 2010; Freeman et al., 2020). For example, messages were not displayed on the websites and therefore adolescents had to search for other features and cues besides source credibility, website appearance, or logo (e.g., Metzger et al., 2010; Strömbäck et al., 2020). Nor could adolescents rely on endorsement by other people (e.g., Metzger et al., 2010). Moreover, the participants could not check the content of the message and compare the information with other sources as to its accuracy, fairness, or bias (e.g., Brante and Strømsø, 2018; Strömbäck et al., 2020). Therefore, adolescents could only rely on themselves and the information in the provided messages.

One possible explanation of participants’ failure to notice the editing cues comes from the tripartite model of mind proposed by Stanovich et al. (2011: 373–378). In this model, the mind is divided into the autonomous (fast-thinking, intuitive), the algorithmic (slow thinking, rational), and the reflective mind. The latter embraces general knowledge and beliefs. According to this model, individual differences in rational thinking dispositions are shown in a reflective mind because this type of mind refers to goals and beliefs relevant to the goals. Both algorithmic and reflective minds contribute to so-called mindware that encompasses declarative knowledge and procedural skills. Mindware helps to initiate detection processes followed by inhibitory processes that override System 1 (Stanovich, 2018: 432–433). From this point of view, there are two types of judgment errors relating to mindware that can happen: error of comprehension (or knowledge error) and error of application (or process error) (Stanovich et al., 2011: 366; Stanovich, 2018: 433).

The participants might have both judgmenterrors. They either do not have the necessary knowledge or do not know how to use this knowledge. Stanovich et al. (2011: 369) highlight that mindware is a very special subset of several skills and knowledge, such as probability, scientific reasoning, formal and informal reasoning, evaluation skills, examining possibilities, and avoiding myside thinking. The autonomous set of systems, on the contrary, includes behavioral regulation by emotions, implicit learning, and overlearned associations. It seems that adolescents only have the autonomous set present and available. In the case of mindware, they have not created it yet or they might have inadequately learned it (Stanovich, 2018: 440).

In both scenarios, the implication should be focused on learning declarative knowledge or correcting inadequately learned mindware. This is a very important step in prevention or intervention because the availability of mindware is the very key parameter that stands out in the concept of individual differences in heuristics and biases tasks (Stanovich et al., 2011: 379–380). We could see it as the first phase in a 3-stepped model of applying mindware into action. Without knowledge of mindware, one cannot apply this knowledge to practice. And we could start by clarifying the concepts connected to credibility because as Hilligoss and Rieh (2008: 1468–1469) reported that there are at least 5 distinct conceptualizations of credibility by people: truthfulness, believability, trustworthiness, objectivity, and reliability. Then we might introduce cues for online assessment, such as content cues (refers to content itself), peripheral source cues (such as institution, reputation, affiliation), and peripheral information object cues (such as appearance and presentation of the information) (e.g., Hilligoss and Rieh, 2008; Brante and Strømsø, 2018; Park and Kwon, 2018; Strömbäck et al., 2020). Our research also confirms how important different cues are for the adequate assessment of health online messages. Context- and social-free messages are hard to evaluate because a great number of cues are missing. When we help adolescents to become sensitive to cues, they will direct their attention to these cues, as prominence-interpretation theory states (Fogg, 2002: 722–723). Thanks to this attention-grabbing process they can proceed then to evaluate the credibility. The basic tenant in the prominence-interpretation theory says that both these processes are important to judge online information and if one is missing, the users will not be able to evaluate the credibility. Spotting editing cues could later become adequate cognitive heuristics that could help to appraise online health messages effectively, accurately, and correctly (Metzger et al., 2010: 433; Freeman et al., 2020: 219).

The second error regarding mindware relates to errors of process (Stanovich et al., 2011: 366; Stanovich, 2018: 433). Stanovich et al. (2011: 374–375) distinguish between Type 1 processing (heuristics) and Type 2 processing (analytic and reflective). Heuristics are defined as autonomic since they are carried out autonomously and without deliberate cognitive effort, similar to Kahneman (2019). The main function of Type 2 processing is to deactivate heuristics processing and to become engaged in higher cognitive processes. This could be the second reason why the adolescents in this research were not so successful in distinguishing health messages. Type 2 processing was not successful in switching off automatic heuristics processing, Type 2 did not override Type 1 processing. Thus, even if the adolescents had relevant mindware, they used superficial and unsophisticated strategies, such as intuition or other heuristics, in giving their trust to the presented health information; this has previously been confirmed in research (e.g., Freeman et al., 2020). Moreover, based on the credibility assessment model, it is very important what kind of heuristics people have developed or possess because these are then widely used across different sources and contexts (Hilligoss and Rieh, 2008: 1479–1480).

We did not ask participants whether they had any knowledge about trustworthiness or message credibility, nor were they asked to explicitly name the processes used during the evaluation of the trustworthiness of online health messages. However, in recent research (e.g., Gray et al., 2005; McGrew et al., 2018; Tamboer et al., 2022) adolescents admitted that they evaluate online health messages based on the trial-and-error method and they were aware of the need for improving their health literacy skills. It seems that adolescents might be aware of their limits in assessing the trustworthiness of online information and they also might have a need for cognition after all.

Other interesting results concern sensitivity which was computed as the trustworthiness of true messages minus the trustworthiness of fake messages. We found that sensitivity did not correlate with media literacy, but weakly correlated with concrete scientific reasoning and analytical thinking. These concepts were carefully chosen based on previous research, and we supposed that they would help in the evaluation process. However, media literacy as a widely accepted concept for media consumers did not meet our expectations. This could be a result of the used tool, which was a self-reported scale (compared to the other two instruments that were performance tests) and that it focuses on the analysis of media in general. Moreover, in this study, we used informative health messages on the positive effect of fruits and vegetables. In other research on media literacy and health behavior, media literacy was linked to the substance use behavior or risky behavior (Primack et al., 2006: 469; Xie et al., 2019: 153). This could be another explanation for not finding a relationship between sensitivity to health messages and media literacy. Media literacy seems more important in risky behavior and in deciding what to avoid rather than in promoting healthy behavior. Furthermore, informative health messages were not intentionally worded to help adolescents solve their possible health issues. Adolescents probably did not connect to the information as they did not need it.

Two other individual characteristics were related to cognitive abilities, namely analytical thinking and scientific reasoning. Both might contribute to better discerning online health messages (e.g., Čavojová and Ersoy, 2020; Pennycook et al., 2020). In this research, adolescents were poor analytical thinkers, because only 38% of items were answered correctly. However, they were better in scientific reasoning, with almost 65% of correctly answered items. This was corroborated by a stronger relationship of scientific reasoning to sensitivity compared to analytical thinking, even though both correlations were small as to effect size. Analytical thinking seems to help in evaluating both content and format of health messages (fake, true, and edited messages) when an individual needs to decompose the whole into parts in order to evaluate these parts as well as the whole, to find disrupting parts of the message. On the other hand, in discerning fake vs. true health messages scientific thinking helps to find evidence to evaluate these messages and to search for content evidence.

Nonetheless, the adolescents were not keen on using cognitive skills since applying heuristics enables them to spend less energy and sources. “Humans are cognitive misers because their basic tendency is to default to processing mechanisms of low computational expense” (Stanovich, 2018: 424). The second reason why people hinder themselves from applying cognitive abilities is that heuristics must be overruled by some other previously received information or previously learned rule, as was previously stated in the mindware section. If there is no other information or another rule, then obviously one cannot proceed with override.

There is another possible explanation for these results. According to a reflective mind, one needs to have goals and beliefs relevant to this goal. It means that people also need to be motivated and have the self-esteem to apply Type 2 processing and override Type 1 processing (e.g., Stanovich et al., 2011; Stanovich, 2018). Motivation is also mentioned in other models (such as the elaboration likelihood model by Petty and Cacioppo, 1986) or along with credibility assessment (e.g., Metzger et al., 2003; Freeman et al., 2020; Tamboer et al., 2022).

As adolescents are frequent users of the internet, we usually expect that they already know how to approach and appraise online information. The opposite is true, and it seems they have serious gaps in their knowledge. Based on the judgment errors relating to mindware (Stanovich et al., 2011: 366; Stanovich, 2018: 433), we suggest that adolescents should be well trained in procedural knowledge, which means adolescents should know about editorial elements that are characteristic of websites with low-quality content (e.g., Čavojová et al., 2016). On top of that, they should be trained in evaluating online messages (e.g., Petty and Cacioppo, 1986; Metzger et al., 2003; McPherson et al., 2004; Freeman et al., 2020), which means recognizing the cues for evaluation, both central and peripheral, evaluating online messages based on criteria, as well as recognizing problematic messages or misleading cues. This means increasing sensitivity toward cues and enhancing evaluation thinking.

Adolescents should also be trained in analytical thinking and scientific reasoning, as these skills seem to help distinguish false from true health messages. With increased and reinforced knowledge, adolescents can become successful thinkers. They will have two processes and two possible answers available. One that is intuitive and the other one that is a result of analytical thinking. They will better metanalyze their skills, better evaluate their skills and they will not fall for the Dunning–Kruger bias. This effect has been shown in reasoning with intuitive thinkers overestimating their knowledge and analytical thinkers having a more precise estimation of their knowledge (Kruger and Dunning, 1999: 44; Pennycook et al., 2017: 1782–1783). Those who are most biased are the ones that probably neither show propensity to think analytically nor have metacognitive skills to recognize their incompetence (Pennycook et al., 2017: 1782).

However, developing these cognitive advantages is a long-term and demanding process that should be part of the education system. In reality, it seems that the education system does not meet the need for assessment skills and falls short of the requirements of the modern world (Bašnáková et al., 2021: 14–15).

### Limits of the study

This study has several limitations. The sample was limited to high school students, so the sample is biased regarding the proportion of adolescent school structure. Also, the participants had to have access to the internet and be reachable by the agency. There were also twice as many women in our sample as men.

The experiment itself has several drawbacks including ecological validity. In real-life situations, adolescents would probably not read seven health messages in a row to get the information they search for as previously noticed by other researchers (Hansen et al., 2003: e25; Freeman et al., 2020: 221).

Another limitation refers to the internal consistency of the two instruments, namely analytical thinking and scientific reasoning. The results of two different methods (Cronbach’s alpha and McDonald’s omega) were similar and low. This is not surprising since the two instruments are performance instruments that have right and wrong answers, and internal consistency is generally preferred for evaluating scale-based instruments. Other researchers have reported comparable levels of reliability measures for scientific reasoning in the localized version of this instrument (see Čavojová and Ersoy, 2020; Čavojová et al., 2020; Bašnáková et al., 2021). The analytical thinking instrument consisted of four items. In general, lower numbers of items contribute to lower internal consistency (Urbánek et al., 2011). It was also constructed as a combination of selected items from two versions of analytical thinking instruments, namely the numerical version created by Frederick (2005) and the verbal version developed by Sirota et al. (2020). This could contribute to lower internal consistency as well.

We used short health messages with selected vegetables/fruits based on a pilot study. The content of the messages was positive and supportive but might not have elicited enough interest in the adolescents to engage them in the topic. Some adolescents could have been more interested in the health issues and be also more educated in it. Credibility is often connected with the abilities and motivation of the receivers (e.g., Metzger et al., 2003; Freeman et al., 2020). Another potential direction of the future studies could include manipulations with messages on different health related topics.^3^

Health literacy might also prove to be a valuable concept in this regard. Defined as “the acquisition, understanding and application of context-specific knowledge,” health literacy is usually measured on three levels, described as functional, interactive and critical health literacy (Nutbeam, 2009: 304). Higher level of health literacy suggests the greater the autonomy and personal empowerment in health-related issues (Nutbeam, 2009: 304). Moreover, people with lower or limited health literacy are likely to distrust specialist doctors and dentists, but they rely on and put trust in other sources (such as social media, blogs or celebrity webpages, and commercial/corporate sources) that might have dubious information (Chen et al., 2018: 730). The combination of health literacy and media literacy may prove to be the way to go. Some argue that media literacy is not only complementary to health literacy, but one of the factors to increase health literacy would be to increase media literacy (Akbarinejad et al., 2017: 5; Schulenkorf et al., 2021: 5).

As our research shows it is reasonable to explore secondary school students’ perception of online health message credibility. Our results are both disturbing and encouraging at the same time. Adolescents did discern fake messages vis-a-vis true or edited messages. However, almost half of the sample could not differ true from fake messages which is an alarming number. But the hopeful news is that analytical thinking and scientific reasoning seem able to help secondary school students to better discern between fake messages and true messages. Moreover, only clickbait messages stood out among other messages with edited format and content when it came to distinguishing from true messages. Other content and format changes in online health messages (superlatives, authority appeal, bold type, and grammar mistakes) were overlooked by adolescents. These results suggest a way how secondary school students could be better equipped to handle messages in the era of information over-abundance.

## Data availability statement

The datasets presented in this study can be found in online repositories. The names of the repository/repositories and accession number(s) can be found below: OSF https://osf.io/7vjxd.

## Ethics statement

The studies involving human participants were reviewed and approved by the Ethics Committee at Comenius University, Faculty of Social and Economic Sciences. Written informed consent to participate in this study was provided by the participants’ legal guardian/next of kin.

## Author contributions

KG: conceptualization, writing – original draft, data curation, data analysis, and writing – review and editing. RM and VČ: conceptualization, writing – review and editing, and funding acquisition. NS: conceptualization, development of design and methodology, and writing – review and editing. All authors contributed to the article and approved the submitted version.

## Footnotes

1
https://osf.io/7vjxd/

2
http://healthydiet.com/effectofgooseberries

3 We thank for this suggestion to one of the reviewers.
